# Particulate matter exposure induces pulmonary T_H_2 responses and oxidative stress-mediated NRF2 activation in mice

**DOI:** 10.1016/j.redox.2025.103632

**Published:** 2025-04-08

**Authors:** Yuna Jo, Bo-Young Kim, So Min Lee, Jisu Park, Wooseok Kim, Ju A Shim, Jun Hong Park, Jong-Eun Park, Yong-Il Shin, Ji Hyeon Ryu, Changwan Hong

**Affiliations:** a Department of Anatomy, Pusan National University School of Medicine, Yangsan, Republic of Korea; b Research Institute for Convergence of Biomedical Science and Technology, Pusan National University Yangsan Hospital, Yangsan, Republic of Korea; c Department of Convergence Medical Science, Pusan National University School of Medicine, Yangsan, Republic of Korea; d PNU GRAND Convergence Medical Science Education Research Center, Pusan National University School of Medicine, Yangsan, Republic of Korea; e Graduate School of Medical Science and Engineering, Korea Advanced Institute of Science and Technology, Daejeon, Republic of Korea; f Department of Physiology, College of Veterinary Medicine, Jeonbuk National University, Iksan, Republic of Korea; g Department of Rehabilitation Medicine, Pusan National University School of Medicine, Yangsan, Republic of Korea

**Keywords:** NRF2, Oxidative stress, Particulate matter, Th2 immunity

## Abstract

**Introduction:**

Particulate matter (PM) is a harmful air pollutant associated with respiratory and cardiovascular diseases, but its effects on adaptive immunity are poorly understood.

**Objectives:**

This study investigates the role of NRF2 in T cells in mediating immune and pulmonary responses to long-term PM exposure, highlighting its impact on inhalation toxicity.

**Methods:**

To establish a mouse model of lung injury induced by PM exposure, C57BL/6 mice were intranasally administered 20 μg/kg PM_10_ or PM_2.5_ daily for 16 weeks. Lung injury parameters were analyzed in bronchoalveolar lavage fluid (BALF), plasma, and lung tissue. Changes in the proportion of immune cells in the lymph nodes and spleen were analyzed.

**Results:**

Mice exposed to PM for 16 weeks showed severe lung damage, such as inflammatory cell infiltration, thickened alveolar walls, and increased oxidative stress and apoptosis. PM exposure also increased collagen and fibronectin levels, indicating tissue remodeling. Immune cell analysis revealed reduced B cell expansion, increased IL-4-producing CD4^+^ T cells, and decreased IFN-γ- and TNF-α-producing CD4^+^ T cells, accompanied by higher T_H_2 cytokines and plasma IgE and IgG1 levels. PM activated the NRF2 pathway, skewing immune responses toward T_H_2 differentiation, which worsened lung inflammation.

**Conclusions:**

These findings highlight how PM exposure disrupts immune balance and exacerbates conditions like asthma and chronic obstructive pulmonary disease by promoting T_H_2-driven inflammation through NRF2 activation.

## Highlights

• Smaller PM intensifies lung inflammation and fibrosis, exerting more severe effects on pulmonary health.
• PM exposure alters immune cell composition, reducing B cells while increasing T cells and CD4:CD8 ratio.
• PM suppresses T_H_1 responses and promotes T_H_2-related cytokines, shifting overall immune response.
• PM exposure elevates T_H_2 cytokines, IgE, and IgG1 levels, indicating T_H_2 polarization, especially with PM_2.5_.
• PM upregulates NRF2 and its downstream genes, promoting T_H_2 differentiation and pulmonary inflammation.

## Graphical abstract

**Figure:**
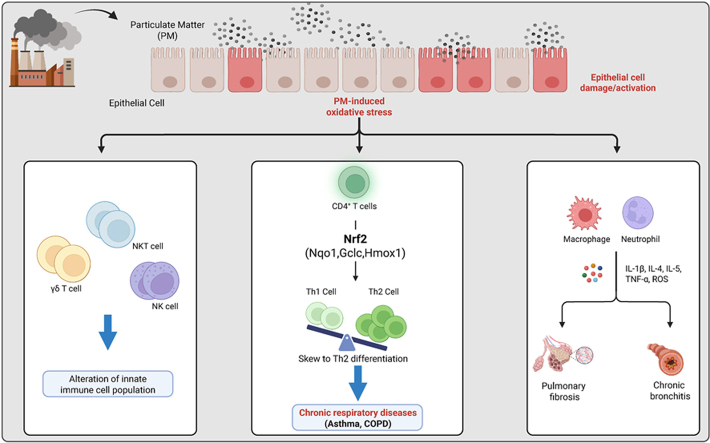

## 1 Introduction

Particulate matter (PM) is among the most hazardous air pollutants, with most of the global population exposed to levels exceeding the safety standards recommended by the World Health Organization (WHO) [1]. “Coarse” (PM_10_; particles <10 μm in aerodynamic diameter) and “fine” (PM_2.5_; particles <2.5 μm in aerodynamic diameter) particles are of concern [2]. While PM_10_ and PM_2.5_ penetrate the lungs via the respiratory tract, PM_2.5_ can reach the circulatory system, leading to multiple systemic injuries [3].

Upon inhaling PM, reactive oxygen species (ROS) and cytotoxic metabolites are generated on the epithelial layer, inducing inflammation by recruiting neutrophils and macrophages [[4], [5], [6]]. PM_2.5_ induces pulmonary inflammation by inducing oxidative stress [7] and inflammatory cytokine release, particularly tumor necrosis factor (TNF)-α, interleukin (IL)-1β [8], IL-6, and granulocyte-macrophage colony-stimulating factor (GM-CSF) [9] by macrophages [10]. Additionally, PM_10_ induces neutrophil infiltration into the lung and upregulates proinflammatory factors, including ROS and IL-8 [11]. Chronic PM exposure is directly associated with the incidence and progression of respiratory diseases, including asthma, chronic obstructive pulmonary disease (COPD), and lung cancer [12].

Interest in the harmful effects of PM on the immune system has recently increased [13]. PM accumulates within the macrophages in lung-associated lymph nodes (LN), causing long-term immune surveillance impairment [14]. Although PM directly impacts immune cell functionality and alters the lymphatic architecture, most available data focuses on the innate immune system. While respiratory diseases and allergies influenced by PM are regulated by adaptive immune cells [15,16], the role of PM in the adaptive immune system remains poorly understood.

Nuclear factor erythroid 2-related factor 2 (Nrf2), a master regulator of cellular redox homeostasis, plays a pivotal role in T cell lineage commitment through modulation of antioxidant response pathways. While prior investigations have focused on Nrf2-dependent oxidative stress mitigation in innate immunity [17,18], this study uncovers its T cell–intrinsic regulatory mechanism governing PM-induced adaptive immune responses. We identify Nrf2 as a direct regulator of CD4^+^ T helper cell polarization, demonstrating its dose-dependent suppression of Th1/Th2 bias during chronic PM exposure. These results establish a novel immunotoxicological axis linking environmental pollutant sensing to adaptive immune plasticity.

## 2 Materials and methods

### 2.1 Animal experiment and PM exposure

All animal experiments were approved by the Institutional Animal Care and Use Committee of Pusan National University Yangsan Hospital (2022-030-A1C0) and Pusan National University (PNU-2021-2975 and PNU-2022-3183). Six-week-old male C57BL/6 mice were purchased from Koatech Animal Breeding Center (Pyongtaec, Korea) and housed in a pathogen-free containment facility with food and water *ad libitum*. The body weights of the mice were measured weekly for four months. The B6 Nrf2^−/−^ mice [19] were provided by Dr. M. Joo (Pusan National University). The Nrf2^−/−^ mice used in our study are whole-body knockout models. Nrf2-transgenic (Tg) mice with T cell-specific Nrf2 overexpression were generated by ligating murine Nrf2 cDNA into human CD2 (hCD2) enhancer promoter-based vectors, which were then transfected into fertilized B6 oocytes [20].

The PM_10_ (PM_10_-like; ERM-CZ100) and PM_2.5_ (PM_2.5_-like; ERM-CZ110) were purchased from European Reference Materials (B-2400, European Commission, Geel, Belgium). The detailed manufacturing processes of PM samples are specified in their corresponding certification reports [21,22].

**Fig. 1:**
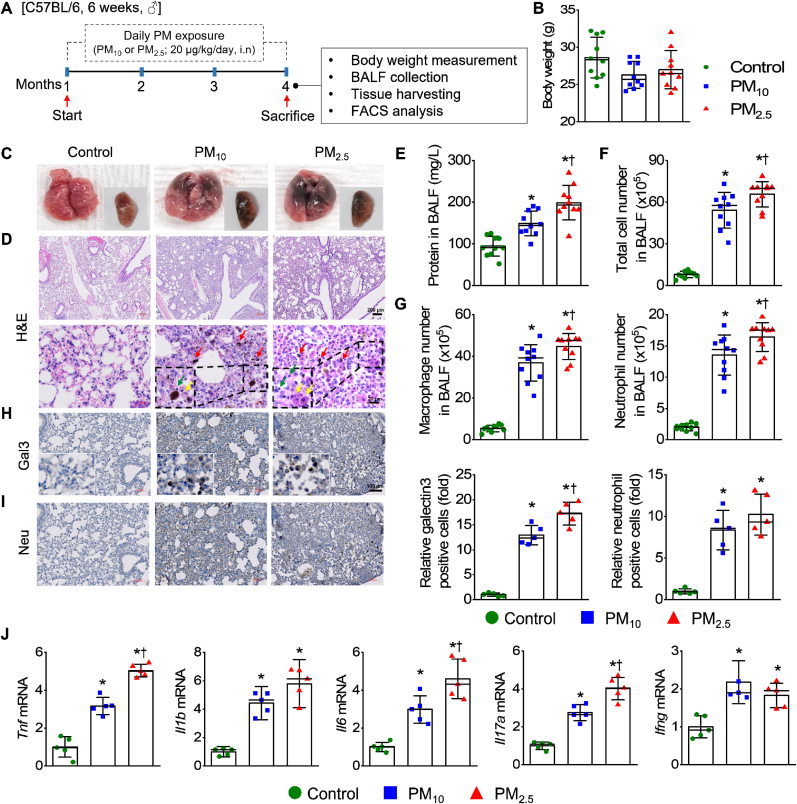
PM exposure results in pulmonary injury in mice. (**A**) Schematic of establishment of PM-induced lung injury mouse model. Male C57BL/6 mice (6 weeks old) were treated with 20 μg/kg PM_10_ or PM_2.5_ in 20 μL PBS daily via intranasal instillation (i.n.) for four months; control mice were treated with PBS. (**B**) Total body weight measured at the end of the 4-month exposure period. (**C**) Gross morphology of the mouse lung. (**D**) H&E staining of the lung tissues; yellow and green arrows: macrophage and lymphocyte infiltration, respectively; red arrows: PM particle penetration into the lung tissues. The dashed-line boxes delineate regions of interest characterized by prominent infiltration of inflammatory cells, including macrophages and lymphocytes, as demonstrated in the enlarged lower panel. Scale bar: 200 μm (upper panel) and 20 μm (lower panel). (**E**) Protein concentration in BALF. (**F**) Total cell and (**G**) macrophage and neutrophil counts in BALF. Lung sections stained with antibodies specific for (**H**) macrophages (Gal3) and (**I**) neutrophils (Neu). Quantification of positive cells of Gal3 (**H**) and Neu (**I**) in each experimental group. (**J**) mRNA levels of *Tnf*, *Il1b*, *Il6*, *Il17a*, and *Ifng* in lung tissues (*n* = 5–10 per group). Scale bar: 100 μm. *∗p* < 0.05 compared with control. ^†^*p* < 0.05 compared with PM_10_.

Mice were randomly divided into three groups (*n* = 10 per group): control, PM_10_ exposure, and PM_2.5_ exposure. To assess NRF2's role in PM-induced lung injury and immune response, wild-type (WT), Nrf2^−/−^, and Nrf2Tg mice were used (*n* ≥ 4 per group). Mice were anesthetized using isoflurane (Hana Pharm Co., Ltd., Hwaseong, Korea) at 2 % for induction and 1.5 % for maintenance in a mixture of N_2_O and O_2_ PM exposure was induced through daily intranasal instillation of 20 μg/kg PM_10_ or PM_2.5_ suspended in 20 μL of phosphate-buffered saline (PBS) for four months; the Nrf2^−/−^ and Nrf2Tg studies were conducted over ten weeks. During PM injection, control mice were treated with PBS (Fig. 1A). To prevent swallowing of the solution, mice were positioned on their backs with their heads manually held at 70–90°.

### 2.2 Collection of bronchoalveolar lavage fluid (BALF) and cell counts

BALF was collected, and cells were counted as previously described [23]. The mice were administered a lethal dose of avertin tribromoethanol (Sigma-Aldrich Chemical Co., St. Louis, MO, USA), and the lungs were lavaged with 1 mL of ice-cold PBS via a tracheostomy tube. BALF was centrifuged at 300×*g* for 10 min at 4 °C. The bronchoalveolar lavage cells were counted using a hemocytometer. BALF samples were frozen at −80 °C for further cytokine analysis. BALF protein content was measured using a bicinchoninic acid (BCA) assay kit (Thermo Fisher Scientific, Rockford, IL, USA). Differential cells were attached to slides by cytocentrifugation (Cytospin; Thermo Shandon, Pittsburgh, PA, USA) and stained with Diff-Quick (Sysmex International Reagents, Kobe, Japan).

### 2.3 Enzyme-linked immunosorbent assay

The levels of mouse total immunoglobulin E (IgE; Bethyl Laboratories, Montgomery, TX, USA), Ig Isotyping (Invitrogen, Camarillo, CA, USA), cytokines (TNF-α, IL-1β, IL-6, IL-17, IFN-γ, IL-4, IL-5, and IL-13; R&D System, Minneapolis, MN, USA), 4-HNE (FineTest, Wuhan, China), and fibronection (Abcam, Cambridge, UK), and superoxide dismutase (SOD) (Thermo Fisher Scientific) were quantified using ELISA kits following the manufacturer's instructions. The absorbance was measured at 450 nm using a Tecan Infinite M200 PRO Microplate Reader (Tecan Austria GmbH, Grödig, Austria).

### 2.4 Measurement of total free radical activity in lung tissue

Total ROS/reactive nitrogen species (RNS) levels in lung tissues were quantified using an OxiSelect™ ROS/RNS assay kit (Cell Biolabs, San Diego, CA, USA) following the manufacturer's protocol. Briefly, the lung tissues were homogenized in radioimmunoprecipitation assay (RIPA) buffer (Thermo Fisher Scientific) to obtain tissue lysates, which were subjected to sonication for 1 min on ice and centrifugation at 12,000×*g* for 15 min at 4 °C. Fluorescence intensity was measured using a microplate reader (SpectraMax iD5 Molecular Devices, San Jose, CA, USA).

### 2.5 Histology and immunohistochemistry

Tissue samples from the right lung were immediately fixed in 4 % paraformaldehyde (PFA) solution for 24 h, embedded in paraffin, sectioned to 5 μm, deparaffinized, and rehydrated.

Lung inflammation was analyzed using hematoxylin and eosin (H&E; Sigma) staining, and lung fibrosis was analyzed using Masson's trichrome (MT; Polysciences, Warrington, PA, USA) and Picro Sirius Red (Abcam, Cambridge, UK) staining, following the manufacturer's instructions. The intensities of collagen deposition in the MT- and Picro Sirius Red-stained sections were scored as follows: 0, no collagen deposition; 1, thin collagen layer; 2, collagen cluster; 3, thick collagen layer. Five sections from each sample were examined by imaging under a virtual microscope (Axio Scan. Z1; Carl Zeiss, Jena, Germany). Histological assessment was performed by three blinded independent observers, and the average scores were recorded.

Immunohistochemical detection of galectin-3 (GAL3), neutrophils (Neu), and 8-hydroxy-2′-deoxyguanosine (8-OHDG) in tissue slides was performed by antigen retrieval; endogenous peroxidase was inactivated by 3 % hydrogen peroxide treatment. The slides were washed thrice in PBS and incubated with blocking buffer (10 % normal goat serum in PBS) for 60 min at room temperature. The slides were incubated with anti-GAL3 (Abcam), anti-Neu (Abcam), and 8-OHDG (Abcam) antibodies overnight at 4 °C. They were then incubated with a horseradish peroxidase-conjugated goat anti-rabbit IgG antibody (DAKO, Carpinteria, CA, USA) for 60 min to detect immunoactivity, followed by detection using a DAB solution kit (DAKO). Hematoxylin was used as a counterstain. The stained specimens were visualized under a virtual microscope (Axio Scan. Z1; Carl Zeiss). The average scores of three independent observers blinded to the experimental conditions were recorded.

Terminal deoxynucleotidyl transferase dUTP nick-end labeling (TUNEL) staining was performed to evaluate the degree of apoptosis using the DeadEndTM Fluorometric TUNEL System kit (Promega Corporation, Madison, WI, USA) according to the manufacturer's instructions. Additionally, the lung sections were incubated with 4′,6-diamidino-2-phenylindole (DAPI; 5 μg/mL). Images of TUNEL-positive cells were obtained using a fluorescence microscope (Axio Imager M1, Carl Zeiss).

### 2.6 Quantitative real-time polymerase chain reaction (qRT-PCR) and Western blot

qRT-PCR was performed as previously described [24]. Total RNA was extracted from the left lungs of mice using TRIzol (Invitrogen, Waltham, MA, USA); 2 μg of total RNA was used to synthesize the first cDNA strand using the amfiRivert Platinum cDNA synthesis master mix (GenDEPOT, Barker, TX, USA) according to the manufacturer's instructions. Quantitative PCR was performed using FastStart Essential DNA Green Master (Roche Diagnostics, Mannheim, Germany). Lymph node T cells (LN T) were isolated from Nrf2^−/−^, B6 WT, and Nrf2Tg mice using BioMag goat α-mouse IgG beads (Qiagen, Hilden, Germany). Total RNA was isolated using Ribospin (GeneAll). RNA was reverse transcribed into cDNA using oligo (dT) priming with a Reverse Transcription Kit (GeneAll). qRT-PCR was performed using SYBR Green Master Mix (Bio-Rad) on a Light Cycler 96 Real-Time PCR System (Roche). The relative mRNA levels were normalized to those of *Gapdh* and *Rpl13*. Primer sequences are listed in Table S1. Western blotting was performed as previously described [24]. The details of the primary and secondary antibodies employed in this study are listed in Table S2. Chemiluminescence intensity was detected using Amersham ImageQuant 800 (Cytiva, Marlborough, MA, USA). Each band was quantitatively determined using the ImageJ software (U. S. National Institutes of Health, Bethesda, MD, USA).

### *2.7* *In vitro* T cell differentiation

Naïve CD4^+^ T cells from the LN of WT, Nrf2^−/−^, and Nrf2Tg mice were cultured under T_H_ differentiation conditions for five days. For *in vitro* differentiation, conditioned media were generated as follows: IL-2 (20 ng/mL; Peprotech, Rocky Hill, NJ, USA), IL-12 (100 ng/mL; eBioscience, San Diego, CA, USA), and α-IL-4 (1 μg/mL; BioLegend, San Jose, CA, USA) for T_H_1; IL-2 (20 ng/mL; Peprotech), IL-4 (200 ng/mL; Peprotech) and IFN-γ (1 μg/mL; BioLegend) for T_H_2.

### 2.8 Flow cytometry

Cells were collected from the LN and spleen and analyzed via FACS Canto or Attune NxT. The data were analyzed using FlowJo version 10 (Version 10, Tree Star, Ashland, OR, USA). Antibodies with the following specificities were used for staining: CD8α (53–6.7), CD44 (IM7), NK1.1 (PK136), TCRβ (H57–597), γδTCR (GL3), CD49d (R1-2), IFN-γ (XMG1.2), TNF-α (MP6-XT22), and IL-4 (11B11) (BioLegend); B220 (RA3–6B2) and IL-17 (TC11-18H10) (BD Biosciences); CD4 (GK1.5) (Thermo Fisher; Waltham, MA, USA). The fluorochrome-conjugated CD1d tetramers loaded with PBS-567 and unloaded controls were obtained from the NIH tetramer facility (Emory University, Atlanta, GA, USA). Anti-mouse CD16/32 (2.4G2) was applied to block nonspecific antibody binding. For intracellular cytokine staining, the cells were restimulated for 4 h with PMA (Merck Millipore, Burlington, MA, USA) and ionomycin (Santa Cruz, Dallas, TX, USA) with brefeldin A (Thermo Fisher Scientific) and fixed and permeabilized with IC fixation buffer (eBioscience).

### 2.9 Statistical analysis

Data are expressed as the mean ± standard error of the mean (SEM). One-way analysis of variance (ANOVA) with Bonferroni post-hoc test or Kruskal–Wallis/Mann–Whitney tests were performed after the normality test (OriginPro2020, OriginLab Corp., Northampton, MA, USA). Statistical significance for FACS analyses was performed using GraphPad Prism.

## 3 Results

### 3.1 Long-term exposure to particulate matter (PM) induces pulmonary injury in mice

To evaluate and compare the harmful effects of different sizes of PM on pulmonary injury, mice were exposed to PM_10_ and PM_2.5_ daily for 16 weeks (Fig. 1A). The body weights of the PM-exposed mouse groups were lower than those of the control group; no differences were observed among the experimental groups (Fig. 1B). The lung tissue of the control group showed no apparent histopathological abnormalities or lesions; those of the PM-exposed groups were notably darkened due to PM deposition (Fig. 1C). H&E staining revealed normal pulmonary histology in the control group; in contrast, the PM-exposed group showed extensive infiltration of inflammatory cells within the interstitial and alveolar spaces, thickened alveolar walls, and deep PM penetration (Fig. 1D). The protein levels in BALF were higher in the PM-exposed group than in the controls and considerably higher in the PM_2.5_ than PM_10_ groups (Fig. 1E). The total cell, macrophage, and neutrophil counts in BALF were significantly increased in the PM-exposed mice compared to control mice, with a more substantial increase in the PM_2.5_ group than the PM_10_ group (Fig. 1F and G). Immunohistochemical staining with Gal3 and Neu antibodies revealed greater macrophage and neutrophil infiltration in the lung tissues of the PM exposure groups (Fig. 1H and I), with significantly higher macrophage infiltration in the PM_2.5_-exposed mice (Fig. 1I). Inflammatory cytokine mRNA levels in lung tissues were significantly increased in PM-exposed groups versus the control group (Fig. 1J). Moreover, *Tnfa*, *Il6*, and *Il17a* mRNA levels were significantly higher in the PM_2.5_ group than in the PM_10_ group.

### 3.2 PM induces pulmonary fibrosis in mice

**Fig. 2:**
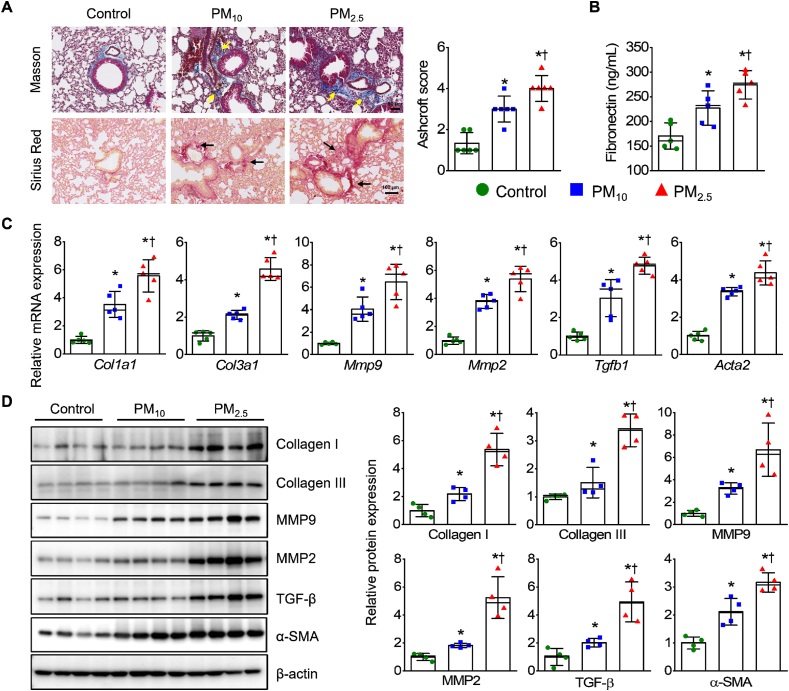
PM promotes pulmonary fibrosis in mice. (**A**) Lung collagen fiber (Masson's trichrome staining) and collagen deposition (Sirius Red staining); yellow and black arrows: collagen deposition. Scale bar: 50 μm (upper panel) and 100 μm (lower panel). Quantification of collagen-fiber depositions. Severity evaluated on a scale from 0 to 3. (**B**) Measurement of fibronectin levels in lung tissues. (**C**) *Col1a*, *Col3a*, *Mmp9*, *Mmp2*, *Tgfb1*, and *Acta2* mRNA levels in lung tissues. (**D**) Abundance of collagen I, collagen III, MMP9, MMP2, TGF-β, and α-SMA in lung tissues. Quantification of protein expression (*n* = 4–6 per group). *∗p* < 0.05 compared with control. ^†^*p* < 0.05 compared with PM_10_.

Collagen deposition was increased in the lungs of PM-exposed mice, as evidenced by MT and Sirius red staining (Fig. 2A). Collagen deposition levels of PM_2.5_-exposed mice were significantly higher than in PM_10_-exposed mice. The fibronectin levels (Fig. 2B) and expression of fibrosis-related genes (i.e., *Col1a*, *Col3a*, *Mmp9*, *Mmp2*, *Tgfb1*, and *Acta2*; Fig. 2C) in lung tissues were also increased in the PM-exposed groups and markedly higher in the PM_2.5_ groups than in the PM_10_ groups. Corresponding increases were observed in the abundance of fibrosis-related proteins (i.e., collagen I, collagen III, MMP-9, MMP-2, TGF-β, and α-SMA; Fig. 2D).

### 3.3 PM induces pulmonary oxidative stress and cell apoptosis

**Fig. 3:**
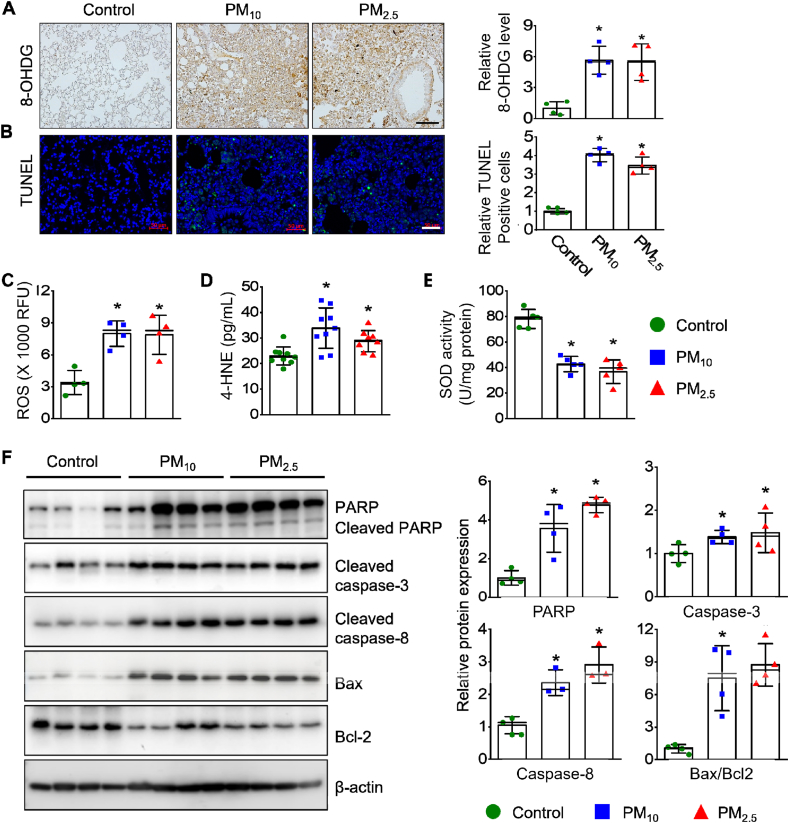
PM exposure induces pulmonary oxidative stress and cell apoptosis. (**A**) Lung sections stained with an antibody specific for 8-hydroxy-2′-deoxyguanosine (8-OHDG). Quantification of 8-OHDG-positive cells. Scale bar: 100 μm. (**B**) Immunofluorescence for TUNEL (green) and DAPI (blue) staining. Scale bar: 50 μm. Bar graphs represent TUNEL (+)/DAPI (+) cells in the lung tissues. **(C**) Levels of reactive oxygen species (ROS) in the lung tissues. **(D**) Measurement of 4-HNE contents. **(E**) Superoxide dismutase (SOD) activity. (**F**) PARP, cleaved caspase-3, BAX, and Bcl-2 abundance in lung tissues. Quantification of protein expression (*n* = 4–9 per group). *∗p* < 0.05 compared with control.

The expression of the oxidative DNA damage marker 8-OHDG (Fig. 3A) and the abundance of apoptotic cells (Fig. 3B) was significantly higher in the lung tissues of the PM-exposed groups than in the control group. Furthermore, levels of ROS and 4-HNE were notably elevated (Fig. 3C and D), while SOD activity (Fig. 3E) was significantly decreased in the lung tissues of the PM-exposed groups compared to the control. The expression of apoptotic markers, including cleaved PARP, cleaved caspase-3, cleaved caspase-8, and BAX, were also significantly increased, whereas that of the anti-apoptotic protein BCL-2 was decreased in the lung tissues of PM-exposed mice (Fig. 3F). These indicators of pulmonary oxidative stress and apoptosis did not differ significantly between the PM_10_ and PM_2.5_ groups.

### 3.4 PM alters the immune cell composition

**Fig. 4:**
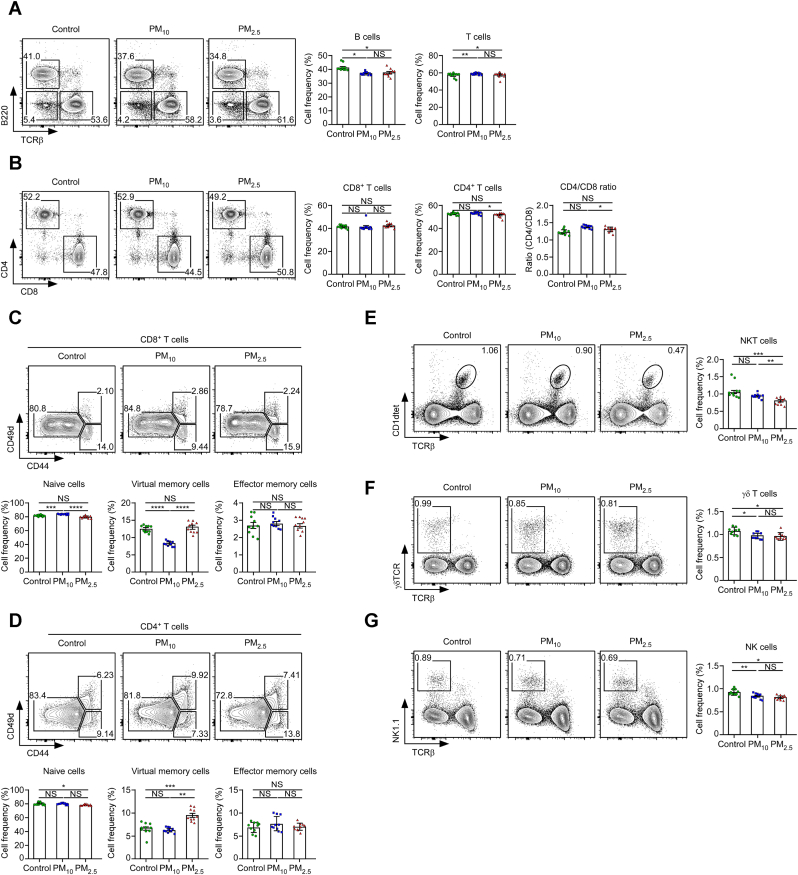
Frequency of immune cells in LN of mice. (**A**) Contour plots of TCRβ/B220 profiles and B and T cell proportions, respectively (left). Summary of B and T cell frequency (right). (**B**) Contour plots of CD4/CD8 profiles and CD4^+^ and CD8^+^ T cell proportions, respectively (left). Summary of CD4^+^ and CD8^+^ T cell proportions in mice (middle). CD4/CD8 ratio in mice (right). (**C**) Contour plots of CD44/CD49d profiles and proportions of CD44^−^CD49d^low^ naïve and CD44^+^CD49d^low/hi^ memory T cells, respectively (top). Naïve- and memory-phenotype analysis of CD8^+^ T cells. Frequency of naïve and memory CD8^+^ T cells in mice (bottom). (**D**) Contour plots of CD44/CD49d profiles and CD44^−^CD49d^low^ naïve, CD44^+^CD49d^low^ virtual memory and CD44^+^CD49d^hi^ effector memory T cell proportions, respectively (top). Naïve- and memory-phenotype analysis of CD4^+^ T cells. The frequency of naïve and memory CD4^+^ T cells in mice (bottom). Contour plots of (**E**, left) TCRβ/CD1dtet, (**F**, left) TCRβ/γδTCR, and (**G**, left) TCRβ/NK1.1 profiles and NKT, γδ T, and NK cell proportions, respectively. Summary of (**E**, right) NKT, (**F**, right) γδ T, and (**G**, right) NK cell proportion. ∗*p* < 0.05, ∗∗*p* < 0.01, ∗∗∗*p* < 0.001, and NS mean not significant.

Because the severity of pulmonary inflammation and injuries in PM-exposed mice was observed, we next determined which immune cells are dominantly affected by PMs. While B and T cells were markedly expanded in the control and PM-exposed groups, PM exposure slightly reduced B cell expansion while increasing T cell abundance without altering their frequency or the B:T ratio (Fig. 4A). The CD4:CD8 ratios were slightly increased in PM-exposure mice compared with control mice; however, greater increases were observed following exposure to PM_2.5_ (Fig. 4B).

PM exposure reduced the proportion of naïve (CD44^−^CD49d^low^) CD8^+^ and CD4^+^ T cells while virtual memory (CD44^+^CD49d^low^) CD8^+^ and CD4^+^ T cell proportions were increased following PM_2.5_ exposure compared with PM_10_ exposure. No differences were observed in the proportion of effector memory (CD44^+^CD49d^hi^) T cells (Fig. 4C and D).

PM exposure also reduced the proportion of NKT cells; however, a greater decrease was observed in the PM_2.5_ exposure group than the PM_10_ and control groups (Fig. 4E). Hence, inhibition of NKT cell proliferation was PM exposure-dependent. Similarly, the proportion of γδ T cells was reduced in PM-exposed mice, particularly within the PM_2.5_ group (Fig. 4F). Additionally, the NK cell frequency was markedly reduced in the PM_2.5_ group (Fig. 4G).

### 3.5 PM exposure suppresses CD8^+^ T cell and T_H_1 immune responses

**Fig. 5:**
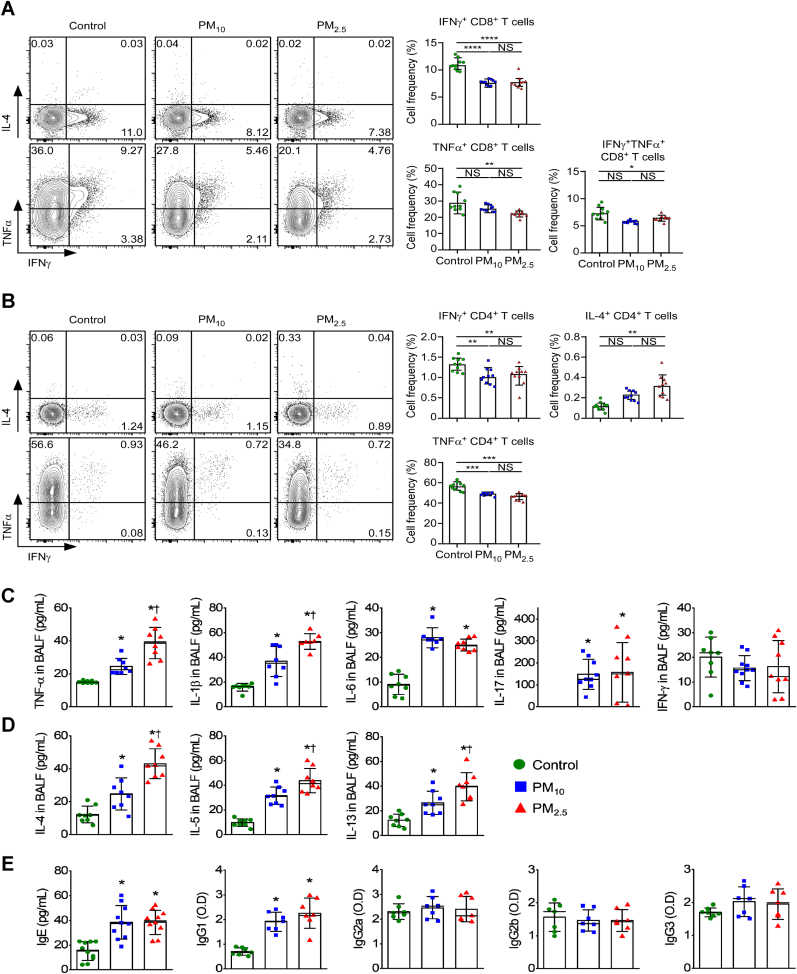
Effect of PM on CD8^+^ and CD4^+^ T cell responses. Lymphocytes isolated from mice were stimulated with PMA/Ionomycin and assessed for IFN-γ, TNF-α, and IL-4 expression in CD8^+^ (**A**) and CD4^+^ T cells (**B**). IFN-γ, TNF-α, and IL-4 profiles are representative of one independent experiment (*n* = 10) (left). Bar graph shows the proportion (%) of IFN-γ, TNF-α, and IL-4-producing CD8^+^ (**A**) and CD4^+^ T cells (**B**). Data represent the mean ± SEM. ∗*p* < 0.05, ∗∗*p* < 0.01, ∗∗∗*p* < 0.001, and NS, not significant. (**C**) Measurement of TNF-α, IL-1β, IL-6, IL-17, and IFN-γ in BALF (*n* = 8). (**D**) Measurement of IL-4, IL-5, and IL-13 in BALF (*n* = 8). (**E**) Plasma Ig concentrations in PM-exposed mice. Sera were collected from three groups (*n* = 7): control, PM_10_ exposure, and PM_2.5_ exposure. Each point represents an individual mouse. *∗p* < 0.05 compared with control. ^†^*p* < 0.05 compared with PM_10_.

To assess how PM affects CD8^+^ and CD4^+^ T cell function during *in vitro* priming, CD8^+^ T cells from control and PM-exposed mice were stimulated with PMA/Ionomycin. In CD8^+^ T cells, exposure to PM markedly reduced IFN-γ and TNF-α expression compared with the control group (Fig. 5A). Correspondingly, PM exposure suppressed the production of IFN-γ and TNF-α compared in CD4^+^ T cells with controls. Meanwhile, IL-4 production was upregulated in the PM_2.5_ group, indicating that PM induced T_H_2-related immune responses (Fig. 5B).

### 3.6 PM exposure induces the T_H_2 immune response

Within BALF, the concentration of the T_H_2 cytokines (IL-4, IL-5, and IL-13) was markedly elevated in the PM-exposed groups, with greater increases in the PM_2.5_ groups (Fig. 5C and D). PM-exposed mice also exhibited significantly increased plasma IgE and IgG1 levels compared with the control group; significant difference did not occur between PM_10_-and PM_2.5_-exposed mice (Fig. 5E). Moreover, the plasma IgG2a, IgG2b, and IgG2 titers did not differ among the groups.

### 3.7 Upregulation of NRF2 via PM exposure skews CD4^+^ T cells toward T_H_2 polarization

**Fig. 6:**
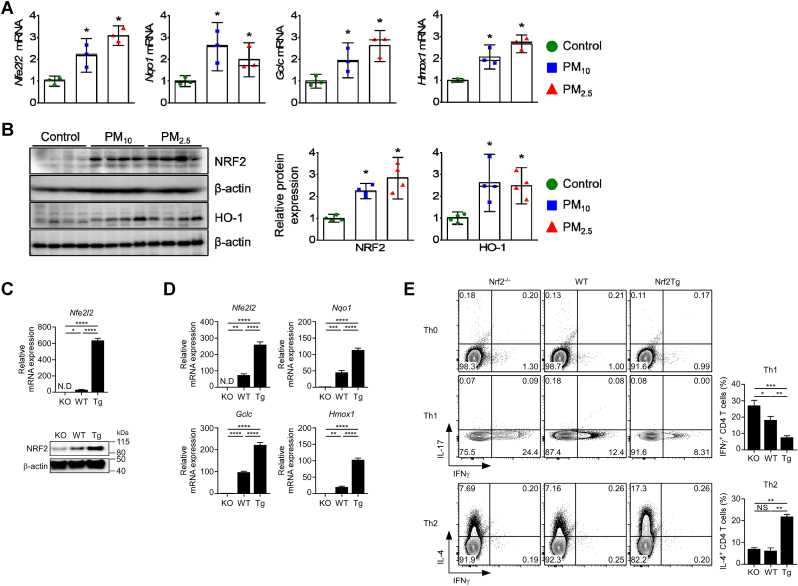
Effect of PM-induced NRF2 upregulation on T_H_1/T_H_2 differentiation. (**A**) *Nfe2l2*, *Gclc*, *Nqo1*, and *Hmox1* mRNA levels in lung tissues (*n* = 3). (**B**) NRF2 and HO-1 abundance in lung tissues. β-actin was used as a loading control. Bar graphs represent the relative protein expression (*n* = 4). *∗p* < 0.05 compared with control. (**C**) *Nfe2l2* mRNA (top) and NRF2 protein (bottom) levels in T cells from WT, Nrf2^−/−^, and Nrf2Tg mice (*n* = 3). β-actin was used as the loading control. (**D**) Expression of *Nfe2l2* mRNA and its target genes in mice (*n* = 5). (**E**) Naïve CD4^+^ T cells were cultured under T_H_ differentiation conditions. IFN-γ, IL-4, and IL-17 levels were assessed using intracellular staining. Contour plots are representative of three independent experiments. ∗*p* < 0.05, ∗∗*p* < 0.01, ∗∗∗*p* < 0.001, and ND, nondetectable.

NRF2 functions as an intracellular sensor of oxidative stress conditions, including those created by PM. The lung tissues of PM-exposed mice showed increased mRNA expression of the antioxidant genes *Nfe2l2*, glutamate-cysteine ligase catalytic subunit (*Gclc*), NAD(P)H quinone dehydrogenase 1 (*Nqo1*), and heme oxygenase-1 (*Hmox1*) (Fig. 6A). Concordantly, the protein expression of NRF2 and HO-1 were markedly increased in PM-treated mice; significant differences were not observed between the PM-treated groups (Fig. 6B).

Additionally, in Nrf2Tg mice, in which NRF2 was overexpressed in T cells under the control of the hCD2 promoter (Fig. 6C), the expression of *Nfe2l2* and its targets *Nqo1*, *Gclc*, and *Hmox1* was upregulated (Fig. 6D). Moreover, overexpression of NRF2 in mice decreased IFN-γ while increasing IL-4 production (Fig. 6E).

These data suggest that Nrf2 activation impairs T_H_1-driven responses, skewing them toward T_H_2 differentiation.

### 3.8 NRF2 directs PM-induced pulmonary injury by regulating CD4^+^ T cell differentiation

**Fig. 7:**
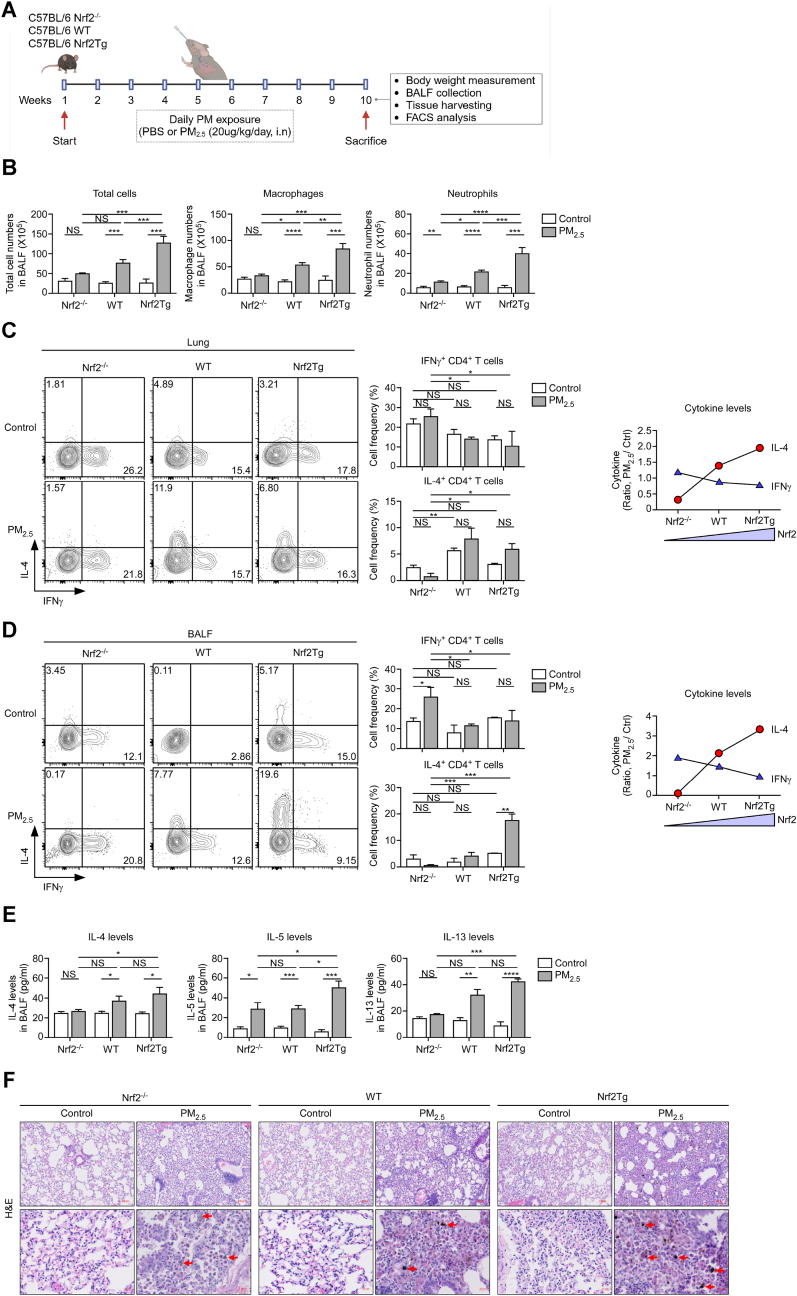
NRF2 deficiency alleviates cytokine responses and symptoms induced by PM_2.5_ exposure. (**A**) Schematic of PM-induced lung injury mouse model. Mice were anesthetized with isoflurane and treated with 20 μg/kg PM_2.5_ in 20 μL PBS daily via intranasal instillation for ten weeks; control mice were treated with PBS (*n* ≥ 4 per group) (**B**) Total cell (left) macrophage (middle), and neutrophil (right) counts in BALF (*n* ≥ 4/group). ∗*p* < 0.05, ∗∗*p* < 0.01, ∗∗∗*p* < 0.001, ∗∗∗∗*p* < 0.0001 and ND, nondetectable. (**C**) Lung and (**D**) BAL cells from mice were stimulated with PMA/Ionomycin and assessed for IFN-γ, and IL-4 expression in CD4^+^ T cells by intracellular staining. IFN-γ and IL-4 profiles are representative of one independent experiment (*n* ≥ 4 per group) (left); bar graph presents the proportion (%) of IFN-γ- and IL-4-producing CD4^+^ T cells (middle); line graph presents the ratio of IFN-γ and IL-4 in PM-induced mice (Ratio: PM_2.5_/control) (right). ∗*p* < 0.05, ∗∗*p* < 0.01, ∗∗∗*p* < 0.001, and NS, not significant. (**E**) IL-4, IL-5, and IL-13 in BALF of PM-induced Nrf2^−/−^, WT, and Nrf2Tg mice (*n* ≥ 4 per group). Data represent the mean ± SEM. ∗*p* < 0.05, ∗∗*p* < 0.01, ∗∗∗*p* < 0.001, and ND, nondetectable. (**F**) Representative images of H&E staining of the lung tissues. Red arrows: areas of PM particle penetration into lung tissues. Scale bar: 100 μm (upper panel) and 20 μm (lower panel).

To investigate the impact of NRF2 expression on immune responses to PM_2.5_, WT, Nrf2^−/−^, and Nrf2Tg mice were exposed to PM_2.5_ intranasally for over two months (Fig. 7A). PM_2.5_-induced Nrf2^−/−^, WT, and Nrf2Tg mice showed no significant differences in body weight (Fig. S1A). However, the BALF protein levels were higher in the PM_2.5_-exposed group than in the controls and considerably higher in Nrf2Tg mice compared to WT and Nrf2^−/−^ mice (Fig. S1B). The total cell, macrophage, and neutrophil counts in the BALF of Nrf2^−/−^, WT, and Nrf2Tg mice were significantly increased in PM-exposed mice compared to control. Notably, these counts were significantly higher in PM_2.5_-exposed Nrf2Tg mice than in Nrf2^−/−^ and WT mice (Fig. 7B). Nrf2^−/−^ mice exhibited elevated levels of IFN-γ in the LN compared to WT mice, whereas Nrf2Tg mice showed a predominance of IL-4 expression in the control group. (Fig. S2). Upon PM_2.5_ exposure, IFN-γ levels in Nrf2^−/−^ mice remained unchanged, demonstrating resistance to PM_2.5_-induced modulation. In contrast, PM_2.5_ exposure did not significantly affect the frequency of IL-4^+^CD4^+^ T cells in any group, as no significant differences were observed between control and PM_2.5_-exposed groups across Nrf2^−/−^, WT, and Nrf2Tg mice. (Fig. S2). In lung tissue and BAL cells, the differences between groups were more pronounced. IFN-γ levels in Nrf2^−/−^ mice remained elevated despite PM_2.5_ exposure. However, PM_2.5_-induced IL-4 levels exhibited a clear positive correlation with Nrf2 expression, with the lowest levels in Nrf2^−/−^ mice and the highest in Nrf2Tg mice (Fig. 7C and D).

The levels of T_H_2-related cytokines, including IL-4, IL-5, and IL-13, were significantly elevated in the BALF of PM_2.5_-exposed Nrf2Tg mice compared to WT and Nrf2^−/−^ mice (Fig. 7E). Additionally, H&E staining of lung tissues revealed normal histology in the control group. In contrast, the PM-exposed Nrf2Tg group showed extensive infiltration of inflammatory cells into the interstitial and alveolar spaces, thickened alveolar walls, and deep PM penetration compared with the WT and Nrf2^−/−^ groups (Fig. 7F).

Thus, Nrf2 modulated the T_H_1–T_H_2 cytokine balance in response to PM_2.5_ exposure. Nrf2 deficiency promoted a sustained T_H_1 (IFN-γ) response, while its overexpression skewed the immune response toward T_H_2 (IL-4), even under environmental stress. These findings highlight the critical role of Nrf2 in regulating immune homeostasis in response to PM exposure.

## 4 Discussion

This study found that exposure to PM_10_ and PM_2.5_ induced pulmonary injury by increasing lung inflammation, fibrosis, oxidative stress, and apoptosis in a mouse model. PM also increased the proportion of IL-4-producing CD4^+^ T cells and T_H_2 cytokines in the BALF and IgE and IgG1 titers in the plasma. PM upregulated Nrf2 and its target genes, resulting in decreased IFN-γ levels and increased IL-4. Collectively, the adverse effects of PM were more pronounced following exposure to PM_2.5_ than PM_10_. Hence, the skewed T_H_2 differentiation caused by PM exposure may contribute to the onset and exacerbation of pulmonary damage and diseases.

Exposure to PM can trigger pulmonary inflammation, leading to inflammatory cell recruitment to the alveoli [[25], [26], [27]] and elevated levels of pro-inflammatory cytokines [26,[28], [29], [30]]. The current study showed an increase in the number of macrophages and neutrophils infiltrating the lungs of mice exposed to PM. Moreover, mice exposed to PM, particularly PM_2.5_, show elevated TNF-α, IL-1β, IL-6, and IL-17 levels in BALF, along with increased *Tnfa, Il1b, Il6, Il17,* and *Ifng* mRNA expression in the lung tissues. Following PM exposure, pro-inflammatory markers such as TNF-α, IL-1β, IL-6, IL-17a, and IFN-γ trigger inflammatory responses in lung tissue, regulate the immune cell functions, and exacerbate lung damage and inflammation [31,32].

This study observed a significant increase in fibrosis-related markers, including collagen deposition, fibronectin levels, and collagen, *Mmp, Tgfb,* and *Acta2* mRNA expression in mice exposed to PM. In the current study, lung tissue fibrosis was notably more severe in mice exposed to PM_2.5_ than PM_10_.

Oxidative stress caused by PM exposure is a key mechanism underlying PM-mediated toxicity and apoptosis [33,34]. PM promotes oxidative stress by decreasing antioxidant enzyme expression [35]. Our results showed that PM reduced the antioxidant capacity by aggravating oxidative stress through increased ROS and 4-HNE production.

Consistent with previous studies [36,37], the current study showed that IFN-γ and TNF-α are secreted by innate immune cells following chronic exposure to PM, suggesting their pivotal role in the associated pathophysiology of pulmonary inflammation. However, there remains an unclear delineation regarding the differentiation of CD4^+^ T cells. In our study, we directly investigated the differentiation patterns of CD4^+^ T cells, previously indirectly confirmed through mRNA expression or BALF cytokine profiling [38], using intracellular staining. We observed a skewing of T_H_2 differentiation, marked by IL-4 rather than IFN-γ expression, in response to PM exposure. The T_H_2 response was further evident based on increased expression of IL-4, IL-5, and IL-13 [39], which was particularly pronounced following PM_2.5_ exposure. Furthermore, examining Ig isotype changes revealed a significant increase in T_H_2 response-associated isotypes, namely IgG1 and IgE, commonly observed in patients with asthma.

The expression of NRF2—a key regulator of oxidative stress [18]—and related antioxidant factors increased upon PM exposure. Oxidative stress induced by PM augmented Nrf2 expression, suggesting a defensive mechanism against oxidative stress by inducing the expression of downstream effector molecules, including antioxidant genes, *Gclc, Nqo1,* and *Hmox1* [17]. The enhanced expression of Nrf2 following PM exposure is also closely linked to T_H_2 cell differentiation [40]. Indeed, IFN-γ expression was markedly increased in Nrf2-deficient CD4^+^ T cells, whereas IL-4 production was enhanced in Nrf2-overexpressing CD4^+^ T cells. Collectively, PM-induced oxidative stress appears to upregulate Nrf2 expression in T cells as part of the antioxidant response, which promotes T_H_2 over T_H_1 responses.

To further investigate the role of Nrf2 in T_H_2 responses and its impact on PM-induced lung pathology, we exposed WT, Nrf2^−/−^, and Nrf2Tg mice to PM_2.5_. Consistent with previous studies, PM_2.5_ exposure disrupted autophagy in lung tissues, while Nrf2 deficiency restored autophagic processes and maintained lung function and histological stability in PM_2.5_-exposed mice [41]. These results highlight the harmful role of Nrf2 in promoting T_H_2 responses during PM exposure, which exacerbates pulmonary inflammation.

The distribution of unconventional T cells, including NKT, γδ T, and NK cells, decreased under chronic exposure to PM. These cells play a pivotal defensive role against pathogens and cancer. Their reduction following PM exposure may be associated with compromised immune function. However, excluding NKT cells, no significant differences were observed in the unconventional T cell subsets between the PM_10_ and PM_2.5_ exposure groups. Additionally, the activity of cytotoxic CD8^+^ T cells significantly decreased upon PM exposure. This indicates impaired protective immune functions correlated with decreased NK, γδ T, and NKT cell proportions. Therefore, PM compromises the survival of NK, NKT, γδ T, and CD8^+^ T cells, with pivotal roles in protective immune functions, and is implicated in the modulation of T_H_2 responses capable of inducing asthma and allergic reactions and severe immune dysregulation. These changes highlight the detrimental impact of PM, particularly in cohorts vulnerable to infection, such as infants and older adults, as well as in patients with chronic respiratory conditions such as asthma or COPD.

## 5 Conclusion

This study provides valuable insights into the complex interplay between environmental pollutants, immune responses, and respiratory health. Our findings reveal novel mechanisms by which particulate matter exposure influences disease development, particularly through its effects on immune cell function and differentiation.

## CRediT authorship contribution statement

**Yuna Jo:** Writing – review & editing, Writing – original draft, Visualization, Investigation, Funding acquisition, Conceptualization. **Bo-Young Kim:** Writing – original draft, Visualization, Investigation. **So Min Lee:** Methodology. **Jisu Park:** Methodology. **Wooseok Kim:** Investigation. **Ju A Shim:** Investigation. **Jun Hong Park:** Investigation. **Jong-Eun Park:** Investigation. **Yong-Il Shin:** Supervision, Conceptualization. **Ji Hyeon Ryu:** Writing – review & editing, Writing – original draft, Methodology, Funding acquisition, Conceptualization. **Changwan Hong:** Writing – review & editing, Writing – original draft, Supervision, Funding acquisition.

## Compliance with ethics requirements

The study was conducted in accordance with the guidelines of the Declaration of Helsinki and approved by the Institutional Animal Care and Use Committee of Pusan National University Yangsan Hospital and Pusan National University.

## Declaration of competing interest

The authors have declared no conflict of interest.

## Data Availability

Data will be made available on request.
