# Supplementary material for: Particulate matter exposure induces pulmonary TH2 responses and oxidative stress-mediated NRF2 activation in mice
Source: Redox Biol. 2025 Apr 8;82:103632. doi: 10.1016/j.redox.2025.103632 (PMC12018062; doi:10.1016/j.redox.2025.103632)
Supplement: Multimedia component 1 [file mmc1.docx]

Supplementary Materials For

Particulate matter exposure induces pulmonary TH2 responses and oxidative stress-mediated NRF2 activation in mice

Jo Y. *et al.*

*Corresponding author. Email: Ji Hyeon Ryu, [wlgus9217@naver.com](mailto:wlgus9217@naver.com)

Changwan Hong, [chong@pusan.ac.kr](mailto:chong@pusan.ac.kr)

**This PDF file includes:**

Fig. S1

Fig. S2

Table S1

Table S2

**A**


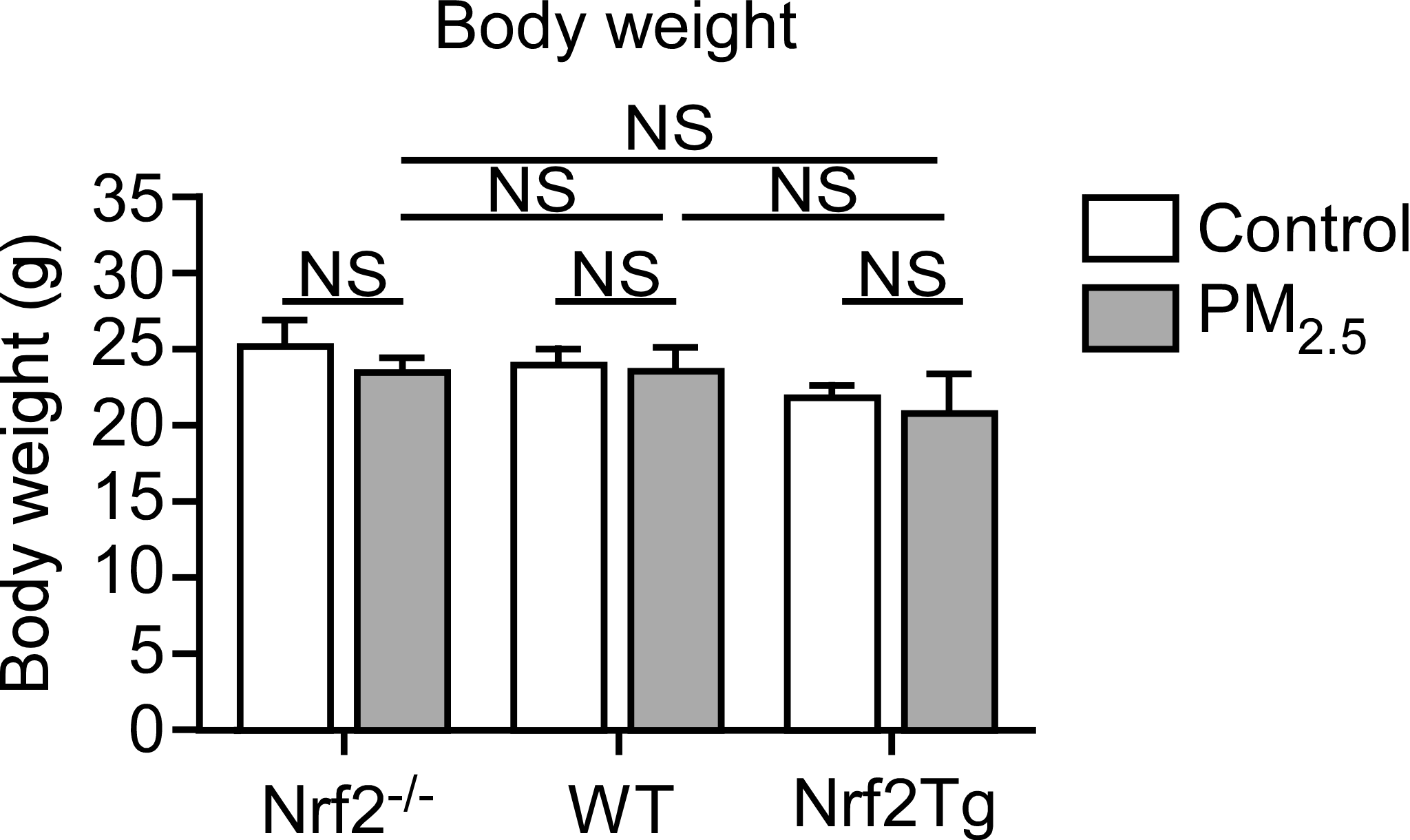

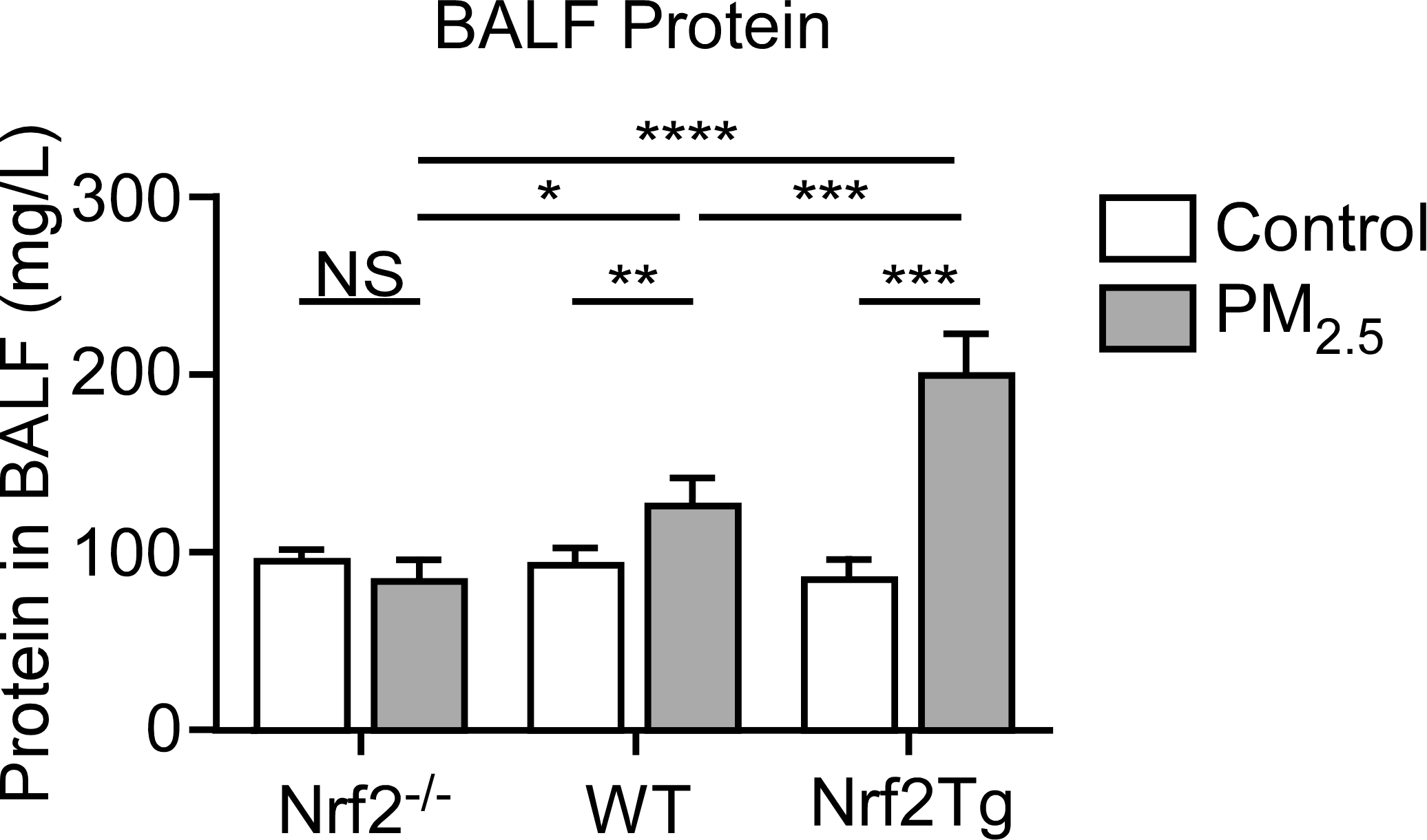


**B**

**Fig. S1. Effects of PM_2.5_ exposure on body weight and BALF protein levels in Nrf2⁻/⁻, WT, and Nrf2Tg mice.**

(A) Body weight comparison among Nrf2^-/-^, WT, and Nrf2Tg mice under control (white bars) and PM_2.5_ exposure conditions (gray bars) at the end of the 10-weeks exposure period. No significant differences (NS) were observed between groups. (B) Total protein concentration in BALF from Nrf2^-/-^, WT, and Nrf2Tg mice under control and PM_2.5_ exposure conditions. Statistical significance was determined using one-way ANOVA with multiple comparisons (**p* < 0.05, ** *p* < 0.01, *** *p* < 0.001, **** *p* < 0.0001, NS = not significant). Data are presented as mean ± SEM (*n* ≥ 4 per group).


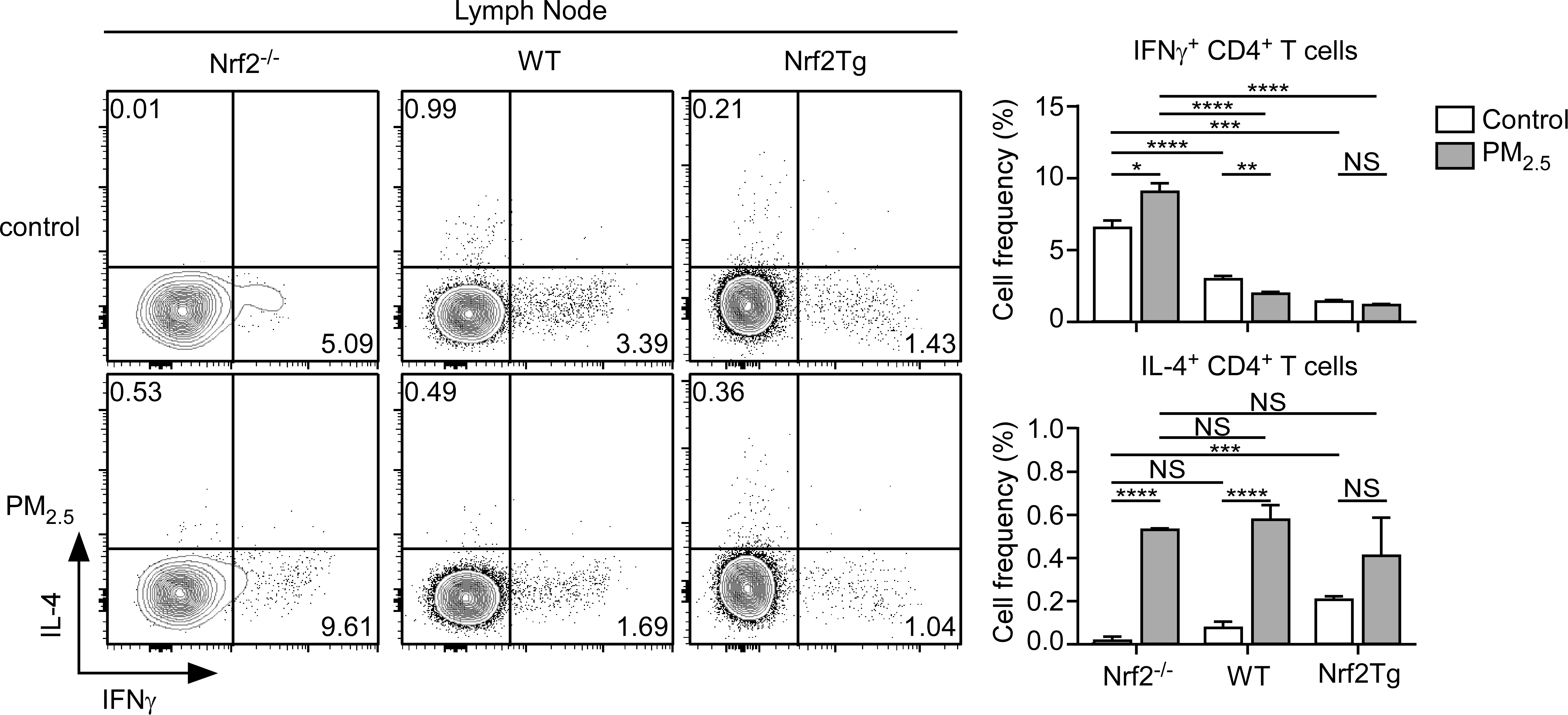

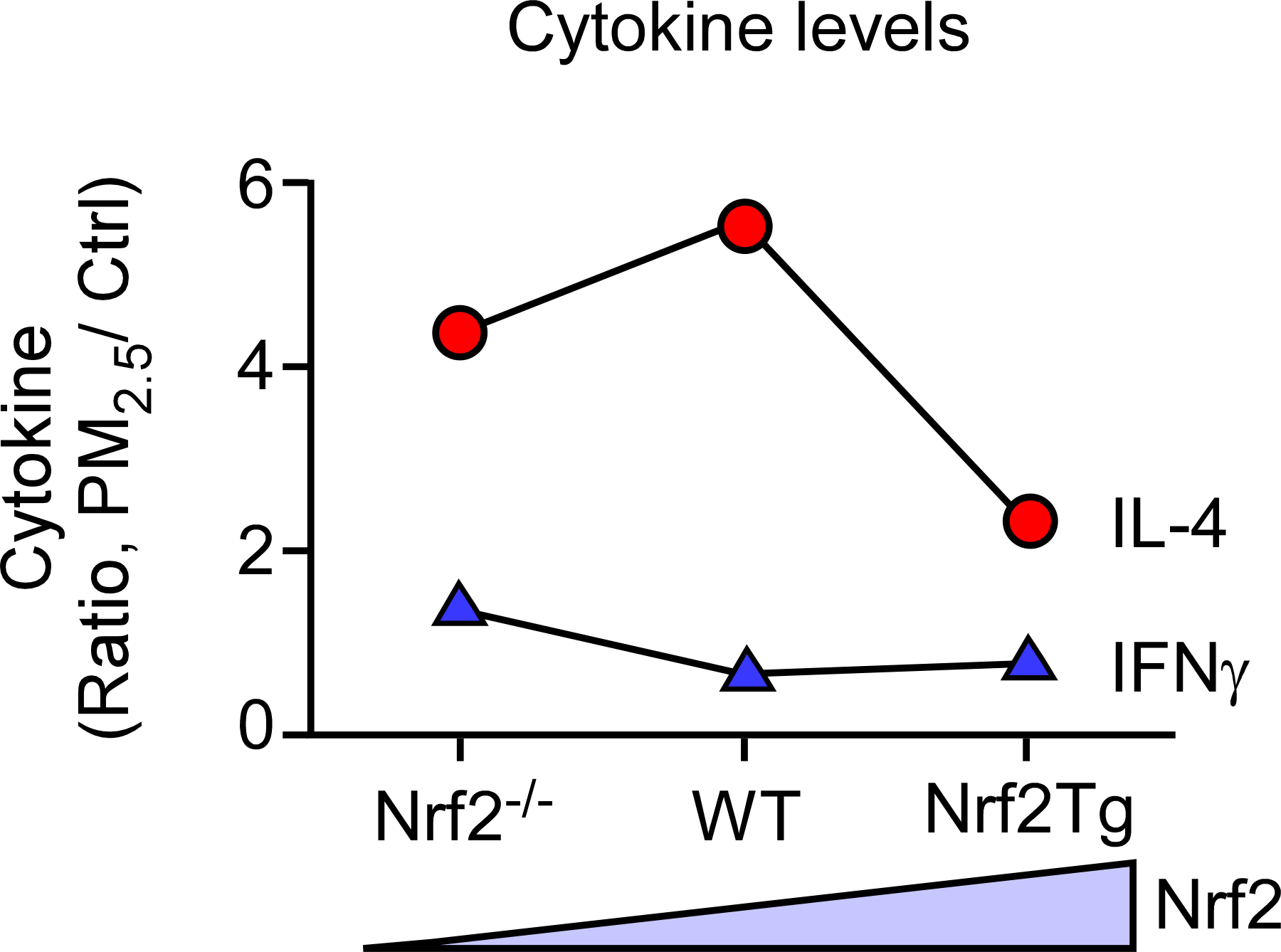


**Fig. S2. Resistance to PM_2.5_-induced modulation and cytokine profiles in Nrf2^-/-^ and Nrf2Tg mice.** Lymph node cells from mice were stimulated with PMA/Ionomycin and assessed for IFNγ, and IL-4 expression in CD4^+^ T cells by intracellular staining. IFNγ and IL-4 profiles are representative of one independent experiment (*n* ≥ 4 per group) (left); bar graph presents the proportion (%) of IFNγ- and IL-4-producing CD4^+^ T cells (middle); line graph presents the ratio of IFNγ and IL-4 in PM-induced mice (Ratio: PM_2.5_/control) (right). Data represent the mean ± SEM. **p* < 0.05, ***p* < 0.01, ****p* < 0.001, and NS, not significant.

**Table S1.** Primer sequences used for qRT-PCR analysis

| **Gene Name** | **GenBank Acc. No.** | **Primer Sequences (5′–3′)** |
| --- | --- | --- |
| *Tnf* | NM_013693.3 | Forward: GAGCAATGACTCCAAAGTAG |
|  |  | Reverse: CCAATTCATCTTGAAATCAC |
| *Il1b* | NM_008361.4 | Forward: TTTGTACAAGGAGAACCAAG |
|  |  | Reverse: TTTCATTACACAGGACAGGT |
| *Il6* | NM_031168 | Forward: CCAATTCATCTTGAAATCAC |
|  |  | Reverse: GGAATGTCCACAAACTGATA |
| *Il17a* | NM_010552.3 | Forward: TTGAGAGAGATCATCGGCATTT |
|  |  | Reverse: CTCACTCTCTGTGGTGTTCTTC |
| *Ifng* | NM_008337.4 | Forward: CTCTTCCTCATGGCTGTTTCT |
|  |  | Reverse: TTCTTCCACATCTATGCCACTT |
| *Col1a1* | NM_007742.4 | Forward: AGACCTGTGTGTTCCCTACT |
|  |  | Reverse: GAATCCATCGGTCATGCTCTC |
| *Col3a1* | NM_009930.2 | Forward: GTGACTCAGGATCTGTCCTTTG |
|  |  | Reverse: GTAGAAGGCTGTGGGCATATT |
| *Mmp9* | NM_013599.5 | Forward: CATCGTCATCCAGTTTGGTG |
|  |  | Reverse: AGGGACCACAACTCGTCATC |
| *Mmp2* | NM_008610.3 | Forward: TGAGCTATGGACCTTGGGAGAA |
|  |  | Reverse: CCATCGGCGTTCCCATAC |
| *Tgfb1* | NM_011577.2 | Forward: GTACCTGAACCCGTGTTGCT |
|  |  | Reverse: GTATCGCCAGGAATTGTTGC |
| *Acta2* | NM_007392.3 | Forward: CATGGCATCATCACCAACTG |
|  |  | Reverse: GCTGGGACATTGAAAGTCTC |
| *Nrf2* | NM_010902.5 | Forward: TAGATGACCATGAGTCGCTTGC |
|  |  | Reverse: TCAGCCAGCTGCTTGTTTTC |
| *Nqo1* | NM_008706.5 | Forward: GCATTGGCCACAATCCACCAG |
|  |  | Reverse: ATGGCCCACAGAGAGGCCAAA |
| *Gclc* | NM_010295.2 | Forward: GCACGGCATCCTCCAGTTCCT |
|  |  | Reverse: TCGGATGGTTGGGGTTTGTCC |
| *Hmox1* | NM_010442.2 | Forward: CACGCCAGCCACACAGCACTA |
|  |  | Reverse: GGCTGTTCGGGAAGG |
| *Rpl13* | NM_016738.5 | Forward: CGAGGCATGCTGCCCCACAA |
|  |  | Reverse: AGCAGGGACCACCATCCGCT |
| *Gapdh* | NM_001289726.2 | Forward: GTGGCAAAGTGGAGATTGTTG |
|  |  | Reverse: TTGACTGTGCCGTTGAATTTG |

*Tnf*, tumor necrosis factor; *Il1b*, interleukin 1β; *Il6*, interleukin 6; *Il17a*, interleukin 17A; *Ifng*, interferon γ; *Col1a*, type I collagen α1 chain; *Col3a*, collagen type III α1 chain; *Mmp9*, matrix metallopeptidase 9; *Mmp2*, matrix metallopeptidase 2; *Tgfb1*, transforming growth factor β1; *Acta2*, α‑smooth muscle actin; *Nrf2*, nuclear factor, erythroid derived 2, like 2; *Nqo1*, NAD(P)H dehydrogenase, quinone 1; *Gclc*, glutamate-cysteine ligase catalytic subunit; *Hmox1*, heme oxygenase 1

**Table S2.** The information of primary and secondary antibodies

| **Primary antibodies** | **Source and concentration** |
| --- | --- |
| Collagen I | Abcam |
|  | 1:1000 |
| Collagen III | Abcam |
|  | 1:1000 |
| MMP-9 | Abcam |
|  | 1:1000 |
| MMP-9 | Abcam |
|  | 1:1000 |
| TGF-β | Abcam |
|  | 1:1000 |
| α-SMA | Abcam |
|  | 1:3000 |
| PARP | Cell signaling |
|  | 1:1000 |
| Cleaved caspase-3 | Cell signaling |
|  | 1:1000 |
| Cleaved caspase-8 | Cell signaling |
|  | 1:1000 |
| BAX | Cell signaling |
|  | 1:1000 |
| Bcl2 | Cell signaling |
|  | 1:1000 |
| β-actin | Sigma-Aldrich |
|  | 1:3000 |
| **Secondary antibody** | **Source and concentration** |
| Goat Anti-Rabbit IgG H&L（HRP） | Sigma-Aldrich |
|  | 1:10000 |

PARP, poly ADP-ribose polymerase; BAX, BCL2-associated X; Bcl2, B-cell lymphoma protein 2
